# Topiramate’s effectiveness on weight reduction in overweight/obese persons with schizophrenia: study protocol for a randomized controlled trial

**DOI:** 10.1186/s13063-017-2162-6

**Published:** 2017-09-20

**Authors:** Miyuru Chandradasa, Layani Champika, Silumini de Silva, K. A. L. A. Kuruppuarachchi

**Affiliations:** 1 Department of Psychiatry, Faculty of Medicine, University of Kelaniya, Kelaniya, Sri Lanka; 2 Colombo North Teaching Hospital, Ragama, Sri Lanka

**Keywords:** Schizophrenia, Antipsychotic agents, Weight loss, Body weight, Anticonvulsants, Randomized trial, Protocol, Sri Lanka, Asia

## Abstract

**Background:**

Schizophrenia is a psychiatric disorder with a higher mortality than that of the general population. Most of the deaths are due to cardiovascular causes and are related to metabolic risks. This risk is due not only to antipsychotics but also to inherent factors of the disorder. Studies in the West have shown topiramate to be effective in schizophrenia to reduce weight gain and for symptomatic control. Whether this is effective for South Asians is not known. It is important because South Asians have a higher risk of metabolic syndrome. We aim to conduct a double-blind, randomized controlled trial comparing topiramate add-on therapy with treatment as usual with antipsychotics in patients with schizophrenia in an outpatient setting in Sri Lanka.

**Methods/design:**

Ninety patients with schizophrenia presenting to the Colombo North Teaching Hospital will be randomized to intervention and control groups equally using permuted block randomization. Patients with comorbid metabolic disorders and taking prescribed weight-controlling medications will be excluded. The intervention group will be prescribed topiramate in addition to their antipsychotics in a predefined dosing regimen targeting a dose of 100 mg per day. The control subjects are to receive a placebo. As the primary outcome, anthropometric measurements including weight, waist circumference, skinfold thickness, and body mass index will be recorded at baseline and monthly during the study period of 3 months. The secondary outcome is the change in symptoms according to the clinician-administered Brief Psychiatric Rating Scale. Assessment of capacity will be performed and informed consent obtained from all subjects. Ethics approval has been obtained from the ethical review committee of the Faculty of Medicine, University of Kelaniya, and the trial has been registered in the Sri Lanka Clinical Trials Registry.

**Discussion:**

In this double-blind, randomized controlled trial, we will attempt to assess the effectiveness of topiramate as an add-on therapy compared with treatment as usual for weight control in patients with schizophrenia. To our knowledge, this is the first such study in South Asia, where metabolic risks are found to be higher than in the West and could have unique ethnic factors related to weight gain in schizophrenia.

**Trial registration:**

Sri Lanka Clinical Trials Registry, SLCTR/2017/003. Registered on 20 February 2017. Universal trial number, U1111-1192-9439.

**Electronic supplementary material:**

The online version of this article (doi:10.1186/s13063-017-2162-6) contains supplementary material, which is available to authorized users.

## Background

Schizophrenia is a psychiatric disorder with positive, negative, affective, and cognitive symptoms. It is associated with a shorter lifespan and higher mortality than that of the general population [1]. Many of the premature deaths among patients with schizophrenia are due to cardiovascular disease [2].

Second-generation antipsychotics are increasingly used for the treatment of schizophrenia [3], probably due to less extra pyramidal side effects being seen when using atypical antipsychotics [4]. However, atypical antipsychotics are associated with more metabolic adverse effects, such as weight gain, than the older typical antipsychotics [5].

The metabolic adverse effects in schizophrenia are only partly due to the use of antipsychotics. Schizophrenia itself is associated with higher metabolic complications and cardiovascular mortality [6]. Many patients with schizophrenia are found to have obesity, fasting hyperglycemia, and other metabolic derangements [2].

Topiramate is an anticonvulsant drug used in epilepsy. It is recognized to act on the brain’s glutamate and gamma-aminobutyric acid (GABA) pathways [7]. There are many recent studies showing abnormalities in glutamate and GABA pathways in schizophrenia, such as *N*-Methyl-d-aspartate receptor involvement [8]. Apart from this, topiramate has been shown to be effective in reducing weight in obesity. It is found to be more effective in reducing weight when the patient has a high baseline body mass index (BMI). The effect of topiramate on weight does not seem to depend on the dose of topiramate or the sex of the person [9]. There are two recognized uses of topiramate in schizophrenia. First, topiramate is used as an augmenting agent when there is inadequate symptomatic control in response to antipsychotic treatment. In addition, it is used to reduce weight in patients with schizophrenia [10].

There have been previous double-blind, randomized controlled studies assessing the effectiveness of topiramate in schizophrenia as an option for reducing metabolic parameters such as weight. Narula and others [11] conducted a randomized, double-blind, placebo-controlled study on topiramate’s effectiveness in reducing olanzapine-associated weight gain and metabolic dysfunction. This study showed that topiramate was effective in preventing olanzapine-induced weight gain. In addition, there was an improvement in clinical parameters, measured using the Positive and Negative Symptoms Scale, associated with the use of topiramate in combination with olanzapine [11, 12]. Apart from this, Afshar and others conducted a randomized, double-blind, placebo-controlled study on topiramate as an add-on medication in schizophrenia to improve the clinical parameters in patients [13]. This study revealed that topiramate is also effective in improving metabolic parameters as a secondary outcome in patients with schizophrenia.

In two recently published meta-analyses of randomized controlled trials on topiramate use as an adjuvant therapy in schizophrenia, the authors found that it was safe and effective for symptomatic improvement and weight reduction [14, 15]. In one of the meta-analyses, the authors considered 16 randomized controlled trials; in the other, the authors considered eight trials for analysis. The number of participants in the studies used for analysis has been relatively small [14, 15]. The published studies have been conducted in Western countries, and we were not able to access any from South Asia. Because the risk of obesity depends on the ethnicity of the individual, topiramate’s effectiveness may differ in different populations. It is found that South Asians are more prone to metabolic syndrome at an early age than the white individuals [16]. Apart from this, there is evidence for different antipsychotics to have variable activity in different ethnic populations [17]. In a recent systematic review concerning the prevalence of metabolic syndrome in the Asia-Pacific region, authors found that in most countries, nearly one-fifth of the adult population was affected, and there is a secular trend of increase [18].

Sri Lanka is a developing nation in South Asia with a population of 21 million people. The Colombo North Teaching Hospital in Ragama is the only tertiary care hospital in Gampaha District, which, with a population of more than 2 million people, is the second most populous district in the country [19]. The 1387-bed hospital has a specialized psychiatric service with inpatient and outpatient facilities. The mental health services in Sri Lanka lack physical as well as human resources, and the psychiatrist-to-population ratio is low [20].

The country-wide prevalence of schizophrenia in Sri Lanka is not known. However, the duration of untreated psychosis in patients with schizophrenia in Sri Lanka has been found to be almost 3 years, which is extremely long compared with more affluent countries of the world [21]. In a previous study conducted in Colombo North Teaching Hospital, a higher prevalence of substance use was found among patients with schizophrenia. In this study conducted in 2013, tobacco, cannabis, alcohol, and betel use were seen among 24%, 20%, 11%, and 9%, respectively, of patients with schizophrenia presenting to the hospital outpatient psychiatry clinic [22]. In a later study conducted in 2014 with a larger sample of patients with schizophrenia at the same hospital, researchers found that 43% of the 400 study participants were cigarette smokers, and 88% of them were dependent on nicotine [23]. Betel chewing has been seen at a higher rate among patients with schizophrenia in this setting, which could predispose them to health risks such as oropharyngeal carcinoma [24]. In addition, drivers with schizophrenia and other psychotic disorders treated with psychotropic medications at the Colombo North Teaching Hospital were detected to have more collisions and less earnings per day than a control group [25]. Because community outreach services are at a minimally developed level, many patients with schizophrenia presenting to this hospital are prescribed intramuscular depot antipsychotics. In a study conducted among 481 outpatients with schizophrenia in 2007, 23% had satisfactory compliance, and the compliant patients had low relapse rates at 15%, compared with 65% of the poorly compliant group, during a period of 1 year [26].

To our knowledge, this is the first study on topiramate use for schizophrenia to be done in Sri Lanka. In addition, it appears that the present study would be the first randomized controlled trial in South Asia on topiramate use for schizophrenia. The findings of this study have the potential to provide better treatment options for patients with schizophrenia and obesity in South Asian countries and worldwide.

The primary objective of the study is to determine the possible effectiveness of topiramate in reducing the weight of patients with schizophrenia. Specifically, we aim to explore sociodemographic factors of participants with schizophrenia, assess the mental state and anthropometric measurements of the participants serially, and compare the effectiveness of topiramate in reducing weight in patients with schizophrenia compared with a placebo.

## Methods/design

**Fig. 1:**
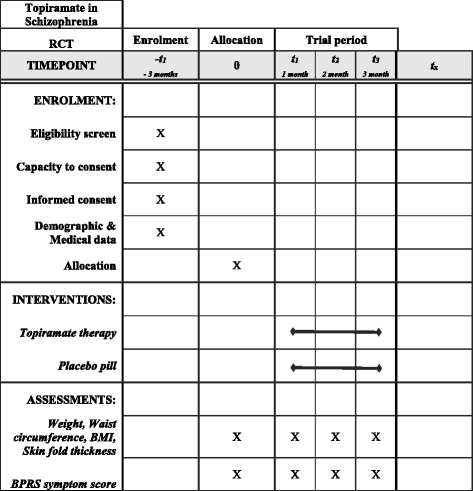
Topiramate’s effectiveness on weight reduction in overweight/obese persons with schizophrenia: enrollment, allocation and assessment schedule. *BMI* Body mass index, *BPRS* Brief Psychiatric Rating Scale, *RCT* Randomized controlled trial

The study will be conducted as a double-blind, randomized controlled trial. The study design is compliant with the Standard Protocol Items: Recommendations for Interventional Trials (SPIRIT) statement [27]. (*See* Additional file 1 for a SPIRIT checklist and schedule of enrollment, interventions, and assessments, as well as the SPIRIT figure shown in Fig. 1.)

### Eligibility criteria

This study will include patients diagnosed with schizophrenia according to the International Classification of Diseases, Tenth Edition [28]. The participants will be recruited from the outpatient psychiatry clinic of the Colombo North Teaching Hospital, Ragama, Sri Lanka. Only adult patients older than 18 years of age and with a BMI greater than the recommended cutoff of 25 kg/m^2^ according to the World Health Organization guidance will be recruited [29].

With regard to exclusion criteria, patients with metabolic disorders such as diabetes mellitus, essential hypertension, and dyslipidemia will not be considered for participation in the study. This is due to the fact that these medical disorders and their treatment could have significant effects on body weight. Any patient on other medications started solely for the purpose of reducing weight will be excluded. Furthermore, any patient with a significant worsening of appetite or difficulty in swallowing will also be excluded from the study. In addition, patients who are pregnant or breastfeeding will not be recruited for the study.

If a recruited participant is being given a new psychotropic medication such an antipsychotic, an anticonvulsant, a mood stabilizer, or an antidepressant or a medical drug such as an oral hypoglycemic during the study period, that participant’s data will be removed from the final analysis. This will be done because the new medication could have a negative or positive impact on the weight of the patient. Therefore, to obscure any undue influence on treatment decisions, the research group will not be involved in decisions regarding management of the participants. Furthermore, if any of the participants are on a medication that is known by medical evidence or by the information contained in the British National Formulary to have serious pharmacodynamic or pharmacokinetic interactions with topiramate, they will be excluded from the study.

### Sample size estimation

Regarding the sample size calculations, in a previous randomized, double-blind, placebo-controlled trial on topiramate use for schizophrenia to alter weight, the mean reduction of weight in the topiramate group was 1.27 ± 2.28 kg. That study recruited 72 patients who were randomized to study and control groups [11]. The researchers in that study found a significant difference in weight change between the groups during a 12-week period. In addition, there was also a significant improvement in the symptom scores of patients with schizophrenia in the above-mentioned trial. The present study is also a randomized, placebo-controlled trial being conducted over a 12-week period. Therefore, we assume that a similar number of participants at the analysis stage, after excluding the dropouts, will be adequate to obtain a significant result for both primary and secondary outcomes, which are described below. On the basis of these facts, we decided to recruit 90 participants for the study, 45 in each group, considering a possible 20% dropout rate during the 3-month period of the assessment.

Another sample size calculation was done using OpenEpi software for studies comparing two means. The inserted values were 95% CI, 80% power, 1:1 ratio of sample sizes between groups, and mean difference of 2.75 kg derived from a meta-analysis of randomized controlled trials on topiramate adjuvant therapy for schizophrenia [14]. The calculated sample size was 40 for each group, which was below the planned sample size calculated using the previous method. We are aiming to obtain a sample size of at least 100, with 50 in each group.

The sample size was determined by targeting the primary outcome of change in weight of patients with schizophrenia. Whether the secondary outcome of change in symptom scores is fulfilled is not essential to achieving the primary goal of the project, because the symptom score assessment is done only to see if there is a possible exacerbation of symptoms due to the addition of topiramate.

### Subject selection

From among the candidates who meet the above criteria, the intervention and control groups will be selected by randomization. The randomization will be conducted by an independent person who has no institutional affiliations with the authors and is knowledgeable based on conducting similar work in the past. The allocation of participants for groups will be strictly done after their recruitment into the study. The allocation generation method will be the restricted method to ensure balanced group sizes. The permuted block randomization method is to be employed with a 1:1 allocation ratio. A block of allocations will have ten participants and five for each group. From among the potential recruits, the participants will be selected using random numbers generated by an online random number generator. A closed, opaque envelope with random numbers is the preferred method of the researchers for randomization. The participants and the researchers will be blinded to the study group to which they belong. The above-mentioned external independent person will carry out the allocations. The staff members who will be attending to routine clinic issues of the patient will be independent of the researchers. The time frame of enrollment, allocation, and assessments is depicted in Fig. 1 in SPIRIT format.

### Blinding

The study is to be conducted as a double-blind, randomized controlled trial as mentioned above. The participants will be blinded to the treatment group to which they are randomized. They will not be informed whether they will be prescribed topiramate, which is the intervention, or the physically similar starch tablet, which is the placebo medication. The physicians will also be blinded to the study arms. The medication will be prescribed under codes specific for each patient.

### Assessment of capacity and informed consent

When the patient visits the clinic, a senior registrar in psychiatry will approach them and provide information regarding the study. Then the patient will be provided with the information sheet in a preferred language. Next, the patient will be given an opportunity to ask questions about the study. Following this, the patient will be accompanied by a research assistant to visit the external consultant psychiatrist in a separate room to assess the patient’s capacity to provide informed consent to participate in the study. If the capacity is decided to be present, the patient will be given the consent form to provide written informed consent.

### Data collection

Subsequently, using an interviewer-administered questionnaire, a senior registrar in psychiatry will gather preliminary information from the participant and direct the participant to a research assistant. The research assistant is a medical doctor specifically trained in obtaining anthropometric measurements from an adult patient. The research assistant will obtain the measurements mentioned in the data record form and will record the initial assessment details. The anthropometric measurements will include height, waist circumference, body weight, and skinfold thickness. In addition, a detailed assessment of eating patterns, daily energy intake, and physical exercise will be conducted. Afterward, the participant will be assessed by a consultant psychiatrist of the research team using the Brief Psychiatric Rating Scale (BPRS), and the symptom score will be recorded [30]. The anthropometric and clinical assessments are to be carried out in the same manner in every monthly visit during the 3-month study period. Details on the age of onset of the disorder, number of relapses, past and current treatments, and the presence of medical disorders will be gathered from the patients and, if necessary, from their relatives for clarification, as well as from the clinic records and past diagnosis cards.

### Intervention

After the clinical assessment is completed, the research assistant will accompany the participant to the randomization room, where the above-mentioned independent person will provide the participant with a code number, which will signify his or her group. Subsequent to this, the participant will be assessed, and the usual medication will be written on the clinic prescription card by medical doctors independent of the research team. Other medications, including antipsychotics, will be continued independent of the study. The participant will be given a prescription according to the predefined dosage under a specific code for the individual patient.

The study group is to be commenced on oral topiramate 25 mg once nightly at the beginning. The dosage is to be titrated up every 2 weeks by 25–50 mg, depending on the tolerability. The targeted level is 100 mg daily in twice-daily doses. Participants in both groups are planned to be screened for any adverse effects pertaining to topiramate therapy. The control group will be provided with a placebo containing starch as a pill that is similar in appearance to the topiramate pill. A number of tablets provided will match the usual tablet count of the intervention group. Adherence is to be monitored via daily medication intake records maintained by the patient and/or a family member. At the end of the study period, each participant and the relevant physician are to be informed of the medication prescribed. After being explained the benefits and drawbacks of continuing topiramate, the patient will be allowed to decide on further topiramate therapy.

All participants are to be informed of adverse effects of topiramate, regardless of their treatment arm, as well as of what measures are to be taken if side effects occur. This medication will be recorded in written form in a separate, closed file, and the medication is to be handed to the participant over a special counter at the hospital dispensary. The medications will be coded specifically so that the physicians involved in the study are unaware of the prescription during the study process.

### Study outcomes

The primary outcome of the study is changes from baseline values in body weight, waist circumference, and/or skinfold thickness (or BMI) at three follow-up time points. The points of measurement are after the first, second, and third months of the trial period. The secondary outcome is the change in the symptom score according to the BPRS assessed at three follow-up time points. The symptom scores are assessed only to determine a possible exacerbation of symptoms, even though worsening with topiramate has not been reported in the past.

### Ethical aspects

All clients who are considered for the study will receive a clear explanation that they have the right to refuse participation in the study and that their participation is entirely voluntary. Service users who decline to participate will be eligible for treatment if applicable and will not be disadvantaged in any manner. The participants will be given a clear explanation that their participation is entirely voluntary and is not part of their routine care at the hospital. All participants have the right to withdraw from the study at any time, and this will be clearly conveyed to them verbally and in writing.

Patients who agree to participate will be given a clear explanation that they may or may not receive the intervention drug, topiramate. Apart from adding topiramate, other management aspects will be similar in both the intervention and control groups. None of the study participants will be provided with any financial reimbursement for participating. The study has ethical clearance from the ethical review committee of the Faculty of Medicine, University of Kelaniya, Ragama, Sri Lanka. The trial is registered in the Sri Lanka Clinical Trials Registry [31].

Apart from topiramate therapy, all other treatment decisions for both groups will be independent of the study. The patients will be assessed by doctors who are not part of the research group for independent opinion and management. However, any addition or omission of any psychotropic or other medication is to be reported to the researchers because this could have a significant impact on the weight of the patient. In addition, the collected information is to be recorded under specific codes and is to be kept confidentially on password-protected institutional computers. Only the principal investigator will have direct access to data, and other researchers will have access to it as necessary for a limited period.

### Data management

The data collected will be entered using Excel software (Microsoft, Redmond, WA, USA) and will be analyzed using OpenEpi software. At the end of the study period, calculations of mean weight differences (primary outcome) and BPRS symptom scores (secondary outcome) will be done at each assessment time point. The means will be compared for differences using independent *t* tests, and other relevant statistical analyses will be carried out.

### Adverse effects

Topiramate has previously been used in patients with schizophrenia in studies conducted in other countries and in day-to-day clinical practice in Sri Lanka for mood symptoms and treatment resistance. If there is any serious adverse effect that has life-threatening consequences or requires hospital admission for management, the participant will be referred for necessary medical expert opinion at the Colombo North Teaching Hospital in Sri Lanka. If a serious adverse effect is reported or observed, the specific code of the individual patient for the trial will be overridden, and necessary information regarding the treatment will be made available to the treating physician. In the event of any clinical presentation that is not considered a known side effect of topiramate, the immediate opinion of the physician or the surgeon will be obtained for the relevant participant. If any of the participants in the study develops a life-threatening adverse effect of topiramate that is not yet known, the trial will be suspended until the probable cause of the adverse effect is known. If it is found to be even remotely related to topiramate, the whole study will be terminated. In a situation where more than 5% of participants develop any intolerable adverse effect, even if mild and not life-threatening, the study will be terminated. The intolerability of the adverse effect will be judged by the subjective opinion of the participants or by their reason for withdrawal from the study.

The recruited patients will be educated verbally and in written form on the common adverse effects of topiramate. If such an adverse effect or any other problematic symptom develops in a participant, he or she can report it to the research team verbally at the clinic or via a phone number provided on the information sheet. The participant will be advised to continue or discontinue the medication, depending on his or her side effects. However, as voluntary participants of the study, the participants have the right to stop taking the study-related medication. Any adverse effect that has a complex presentation or is of significant severity will be referred to the physician or the surgeon for opinion and management. The participants of both study arms will be considered as if they are taking topiramate when they inquire about possible adverse effects, because there would be no or minimal adverse reactions to the placebo tablet and the researchers would be unaware of the treatment arm to which the particular patient belongs.

## Discussion

We aim to investigate the potential effectiveness of topiramate in reducing weight in patients with schizophrenia. If the study reveals any benefit of topiramate in this regard, it will offer a reliable pharmacological option to counter the weight increase in schizophrenia. Many factors, such as negative symptoms, cardiovascular morbidity, adverse effects of antipsychotics, lack of belief in health benefits, lower self-efficacy, and social isolation among patients with schizophrenia, have been found to be associated with low physical activity levels [32]. Therefore, in addition to promoting increased physical exercise in this group, using pharmacotherapy to counter weight increase may be more feasible. Many newer weight-reducing medications are expensive and not available in the government health sector of Sri Lanka. Because topiramate is freely available in the government health sector of Sri Lanka, it would be useful for patients who have a limited spending capacity.

Regarding limitations, topiramate is known to be effective as a mood stabilizer [33]. Therefore, it is possible that topiramate may improve the mood of the study participants with schizophrenia. The mood improvement may indirectly influence the physical activity level of the patient and might indirectly impact the patient’s body weight. The ultimate goal of the study is to find the potential effectiveness of topiramate in reducing weight in schizophrenia. Therefore, topiramate would be useful, regardless of the mechanism, if it helps patients with schizophrenia to counter weight gain.

Sri Lanka and other South Asian countries lack substantial facilities for quality mental health care, and mental health is interwoven with deeply ingrained cultural beliefs [20, 34]. Therefore, appropriate allocation of resources is essential to providing optimal care to patients with major psychiatric disorders such as schizophrenia. Mental health is not considered a priority in resource allocation in Sri Lanka, and thus financial backing for research is limited [20]. As a result, good-quality, locally valid evidence for patient management in mental disorders is limited. Therefore, we hope that this double-blind, randomized controlled study conducted in a limited-resource setting will be a pioneering venture to promote evidence-based mental health care for Sri Lankans.

### Trial status

The trial is registered in the clinical trials registry, and the recruitment of participants has commenced from September 2017.

## Additional file

Additional file 1: SPIRIT 2013 checklist: recommended items to address in a clinical trial protocol. (DOC 120 kb)
